# Comparison of Clinical Outcomes between Hyaluronic and Polylactic Acid Filler Injections for Penile Augmentation in Men Reporting a Small Penis: A Multicenter, Patient-Blinded/Evaluator-Blinded, Non-Inferiority, Randomized Comparative Trial with 18 Months of Follow-up

**DOI:** 10.3390/jcm9041024

**Published:** 2020-04-05

**Authors:** Dae Yul Yang, Hyun Cheol Jeong, Kyungtae Ko, Seong Ho Lee, Young Goo Lee, Won Ki Lee

**Affiliations:** 1 Department of Urology, College of Medicine, Hallym Unversity, Kangdong Sacred Heart Hospital, Seoul 05355, Korea; yang1408@hallym.or.kr (D.Y.Y.); koulich@naver.com (H.C.J.); palindromes@hanmail.net (K.K.); 2 Department of Urology, College of Medicine, Hallym Unversity, Dongtan Sacred Heart Hospital, Hwaseong 18450, Korea; shleeuro@hallym.or.kr; 3 Department of Urology, College of Medicine, Hallym Unversity, Kangnam Sacred Heart Hospital, Seoul 07441, Korea; uroyglee@hallym.or.kr; 4 Department of Urology, College of Medicine, Hallym Unversity, Chuncheon Sacred Heart Hospital, Chuncheon 24253, Korea

**Keywords:** augmentation, filler, hyaluronic acid, men, penis, polylactic acid

## Abstract

Although several types of penile augmentation (PA) fillers have been recently introduced, no long-term follow-up studies have compared them. This study aimed to compare the long-term clinical outcomes of hyaluronic acid (HA) and polylactic acid (PLA) filler injections for PA. Our multicenter, patient-blinded/evaluator-blinded, randomized comparative trial was performed for 18 months after the single injection of fillers. Sixty-seven healthy men reporting a small penis were administered an injection between November 2016 and May 2017. Subjects were divided into the HA group (n = 33) and PLA group (n = 34). At 18 months, the mean penile girths had significantly increased in both groups (each *p* < 0.001). Changes in the mean penile girth of both groups were not significantly different during the study period. Satisfaction levels at 18 months were significantly higher than those at baseline in both groups (each *p* < 0.01). Changes in satisfaction levels did not differ significantly during the study period. Injection-associated adverse events (AEs) occurred in three (9.1%) patients in the HA group and in two (5.9%) patients in the PLA group; no serious AEs occurred. In conclusion, HA and PLA filler injections for PA led to significant augmentation and increased satisfaction. Clinical efficacy and safety were comparable between groups.

## 1. Introduction

The need for safer, more effective, and less invasive treatments has increased in all areas of modern medicine. This is especially true for aesthetic medicine [1]. As aesthetic approaches are almost always performed because of the patient’s wants instead of needs, it is essential to consider the risk-benefits ratio before performing the procedure so that both the patient and physician can choose the safest and least invasive approach.

Soft tissue filler injections have become the main treatment for soft tissue augmentation because they are relatively safe, effective, less invasive, and less expensive than most surgical procedures [2]. In the United States, soft tissue fillers are the second most popular minimally invasive procedure and comprised more than 2.6 million procedures in 2018 [3].

Throughout history, penile size has been a major concern for men as a symbol of masculinity [4]. Many men are dissatisfied with their penile size and desire a larger penis [5]. Therefore, various types of penile augmentation (PA) have been introduced to correct medical, psychological, or aesthetic problems [6]. Soft tissue fillers have been used, but their clinical utility has not yet been clearly demonstrated for PA, and no filler has been approved for PA in the United States [7,8]. Although fillers have been comprehensively studied for use in the face, the penile anatomy, function, and injection techniques are clearly different from those of the face [8,9]. Furthermore, the amount of fillers injected in the penis is much larger than that injected in the face [8,9]. Therefore, the proven clinical utility in the face does not indicate clinical utility in the penis.

Although several types of fillers have been introduced for PA, the ideal filler has not yet been established [7,10,11,12,13,14]. Different types of fillers have varying biochemical and clinical characteristics [8]. Accordingly, it is critical to compare the benefits and risks of each filler when counseling patients about what type to use [8,14]. Nevertheless, to our knowledge, only two studies have compared fillers for PA [7,15], and their follow-up durations (both less than 1 year) were insufficient to prove long-term outcomes.

Hyaluronic acid (HA) and polylactic acid (PLA) fillers are commonly used soft tissue fillers that are regarded as temporary [1,3,8]. For PA, both fillers have been introduced in scientific journals and approved in several countries [7,10,11,15]. However, they have different biochemical characteristics. HA fillers have direct and passive effects, whereas PLA fillers have delayed and bio-stimulatory effects [8].

Our study aimed to compare the clinical outcomes of HA and PLA fillers for temporary PA in a randomized non-inferiority trial. During this comparative trial, the differences in clinical courses of the two fillers were observed for 18 months after injections.

## 2. Patients and Methods

### 2.1. Patients

Healthy men between ages 20 and 66 years who wanted to enlarge their penis due to concerns that it was smaller than normal for an adult male were recruited for our study. The exclusion criteria included the following: any congenital or acquired penile malformation, including concealed penis and Peyronie’s disease; severe phimosis; previous penile surgery, including PA and penile prosthesis insertion; history of psychiatric or psychological disorders, including body dysmorphic disorder; and any chronic major systemic disease, including coagulopathy and anticoagulant use. Men suspected of having a micropenis, which is a normally formed penis that is at least 2.5 standard deviations (SD) below the mean size in stretched length for age, were also excluded because an etiologic evaluation is required for these cases [16].

During the initial examination, a detailed medical history was taken, with a focus on psychiatric and psychological problems. A physical examination, especially of the external genitalia, was also performed. Vital signs were observed and laboratory examinations, including complete blood count, liver function, metabolic function, serum electrolyte, blood clotting function, and urinalysis, were performed to assess the general health status.

The study design and possible filler-related adverse events (AEs) were explained in detail. All patients then signed an informed consent form before filler injection.

### 2.2. Study Design

**Figure 1:**
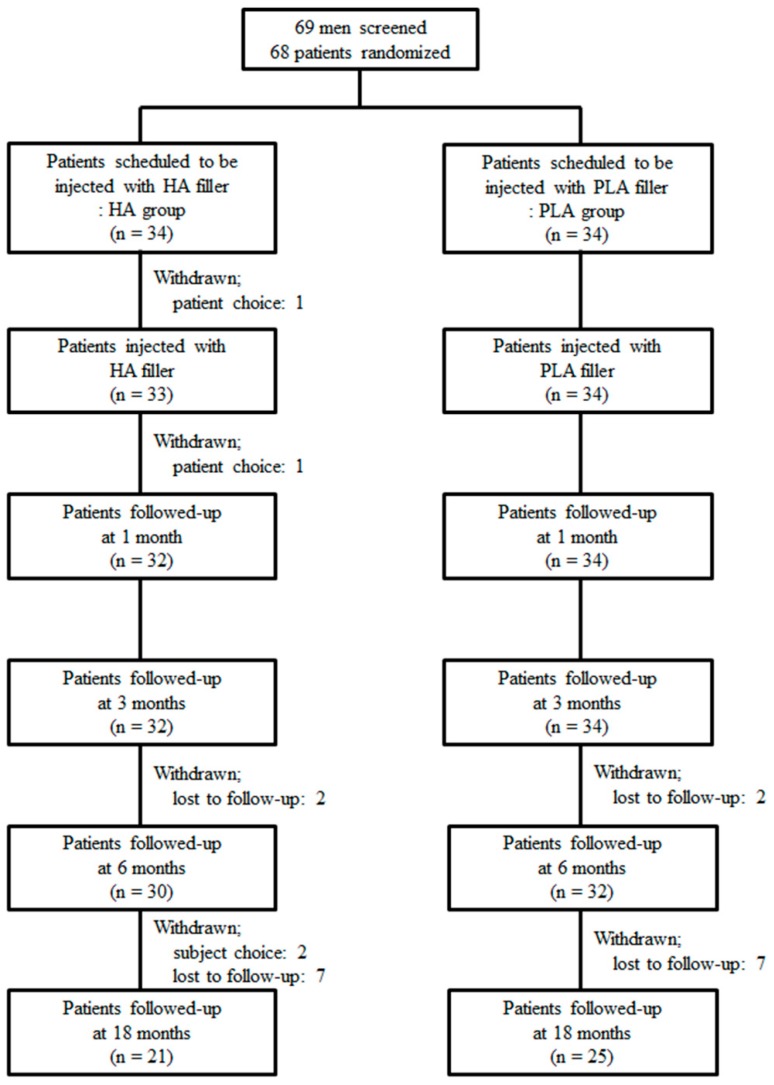
Patients distribution and information. HA, hyaluronic acid; PLA, polylactic acid.

Our multicenter, prospective, patient-blinded/evaluator-blinded, randomized, non-inferiority comparative study comprised a 2-week screening period and 18-month follow-up period after injection (Figure 1). Patients were recruited by an advertisement from three institutions in South Korea between November 2016 and May 2017. The study was approved by the Korean Ministry of Food and Drug Safety (file no. 671-2016) and each institutional ethics committee (file no. 2016-96 at CSHH, 2016-411-S at DSHH, and 2016-08-001 at KSHH). The study was registered at www.clinicaltrials.gov (file no. NCT03153735).

After the screening period, patients at each institution were randomly assigned in a 1:1 ratio to receive HA filler (HA group) or PLA filler (PLA group). Randomization was performed using SAS version 9.4 (SAS Institute Inc., Cary, NC, USA) by an independent biostatistician. The patient-blind procedure was performed with the area below the umbilicus covered. During the evaluator-blind portion of the study, all post-injection assessments at each institution were performed by one trained independent physician who was not involved in screening or filler injection. The patient-blinded/evaluator-blinded status was maintained until the data were finalized.

Penile girth and satisfaction with penile appearance were assessed at baseline and 1, 3, 6, and 18 months after injection. Satisfaction with sexual performance was assessed at baseline and 3, 6, and 18 months after injection. Any AE was reported during either institution visits or immediately when they occurred.

The penile girth measurement was performed in the supine position in the flaccid state without tension and stretching. Penile girth was measured with a measuring tape at the distal shaft (area just below the glans), mid-shaft (area between the proximal and distal shafts), and proximal shaft (area above the peno-abdominal junction). The mean penile girth was defined as the mean of the circumference measurements of the distal, mid-, and proximal shafts. Satisfaction with penile appearance and sexual performance was assessed using a 5-point visual analog scale (VAS). A score of 1 indicated very dissatisfied and a score of 5 indicated very satisfied.

### 2.3. Main Outcome Measures

The primary outcome was the difference in the increase in the mean penile girth of the HA and PLA groups at 6 months after injection. The lower limit of the difference for non-inferiority was determined to be -0.42 cm (HA group compared to PLA group). The 0.42-cm difference was calculated and estimated using the results of previous studies [7,10,11,15]. The primary outcome was measured at 6 months because the initial biochemical changes of both fillers stabilize within 6 months [8].

Secondary outcomes were differences in the changes in penile girth and injection-associated satisfaction of the groups for 18 months after injection.

### 2.4. Safety Assessments

At each institutional visit, medical history, physical examination, and vital signs were assessed by a trained independent physician. Laboratory examinations were performed at baseline, 6 months, and 18 months after injection. Any AEs were reported at onset or at each visit.

### 2.5. Injected Fillers

The HA filler (Hyafilia Impact^®^; CHA Meditech, Co., Ltd., Daejeon, Korea), which has been newly developed for PA, is a transparent gel consisting of 20 mg of cross-linked bi-phasic HA and 3 mg of lidocaine in 1 mL of saline. The PLA filler (PowerFill^®^; REGEN Biotech, Inc., Seoul, Korea), which has been recently developed for PA, is a powder consisting of 10 g of 50 μm PLA microparticles suspended in 3 mL of methylcellulose and carboxymethylcellulose. It should be reconstituted with 7 mL of saline immediately before injection. Both fillers have been approved for PA in several countries.

### 2.6. Injection Method

At each institution, an experienced physician performed the injection procedure. The detailed procedure has been described previously [8]. In summary, the procedure was performed via local anesthesia by injecting lidocaine (Lidocaine HCL Hydrate Injection 2%; Huons, Co., Ltd., Sungnam, Korea) or applying EMLA cream (Emla 5% cream; AstraZeneca Korea, Co., Ltd., Korea). The 17-gauge or 21-gauge injection needle was indwelled on the penile base at the 1–2 o’clock and 10–11 o’clock points. The filler was injected between the dartos and Buck’s fascia using a fanning technique. If necessary, a multiple puncture technique was used at the 3–4 o’clock or 8–9 o’clock points. The injected volume was initially set at 10 to 22 mL, and the actual injection volume was determined according to the penile size and physicians’ experience. After the injection was performed, the patients applied an elastic penile support bandage for 1 day and used antibiotics (first-generation cephalosporin) and non-steroidal anti-inflammatory drugs (NSAIDs) for 3 days. Patients were instructed to massage the penile shaft gently and to abstain from sexual intercourse for 1 month.

### 2.7. Statistical Analyses

Our study was designed with 80% power and a 2.5% level of significance to detect a difference of -0.42 cm in the change in the mean penile girth between groups (HA group compared to PLA group) assuming a common standard deviation of 0.55 cm based on previous studies [7,10,11,15]. At least 27 patients in each group were required for statistical analyses. Finally, 34 patients were included per group considering a dropout rate of 20% during the study period.

Patients who received the filler injection and who were assessed to determine at least one primary outcome measure after injection were included in the main outcome analyses. The last post-baseline observation was carried with the forward approach. To compare differences within or between groups, a repeated measures analysis of variance with a Bonferroni correction and an analysis of covariance including the injected filler volume and baseline mean value as covariates were used. AEs were analyzed for patients who received the filler injection using a Fisher’s exact test.

SPSS for Windows version 21.0 (IBM Corp., Armonk, NY, USA) was used for statistical analyses. The one-sided *p* value for the primary outcome measure was <0.025, which was considered significant; however, other *p* values were two-sided and <0.05 was considered.

## 3. Results

### 3.1. Patients

The baseline demographic and clinical characteristics of patients were similar between groups (Table 1). The mean injected volumes of filler were 16.4 ± 2.7 and 17.7 ± 2.3 mL in the HA and PLA groups, respectively (*p* = 0.031).

Figure 1 shows the patient distribution and information during the study period. A total of 67 patients were injected with fillers. After injection, 62 (92.5%) and 46 (68.7%) were followed-up for 6 months and 18 months, respectively. There was no difference in the follow-up durations of the two groups (*p* = 0.437).

### 3.2. Penile Girth

**Figure 2:**
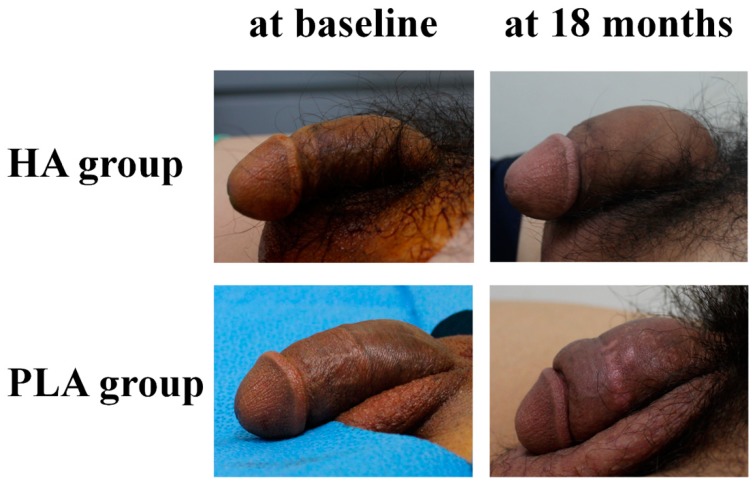
Representative photos of penis before and after hyaluronic acid and polylactic acid filler injections (identification number of patient: R01-23 in the HA group, and R01-03 in the PLA group). HA, hyaluronic acid; PLA, polylactic acid.

Grossly, the penises were smooth, natural, and pliable, and the increase in girth was maintained for up to 18 months after filler injections in both groups (Figure 2). Any irregularity, retraction, lump, or deformity of penis, and migration of fillers were not observed. However, the HA materials were softer and more pliable than PLA materials.

At six months after injection, the mean penile girth increases were 1.91 cm (95% confidence interval (CI), 1.35-2.47; *p* < 0.001) in the HA group and 1.95 cm (95% CI, 1.56-2.33; *p* < 0.001) in the PLA group. The lower limit of the difference for non-inferiority (HA group compared to PLA group) was –0.20 cm, suggesting that the augmentative effect of the HA filler was not lower than that of the PLA filler.

**Figure 3:**
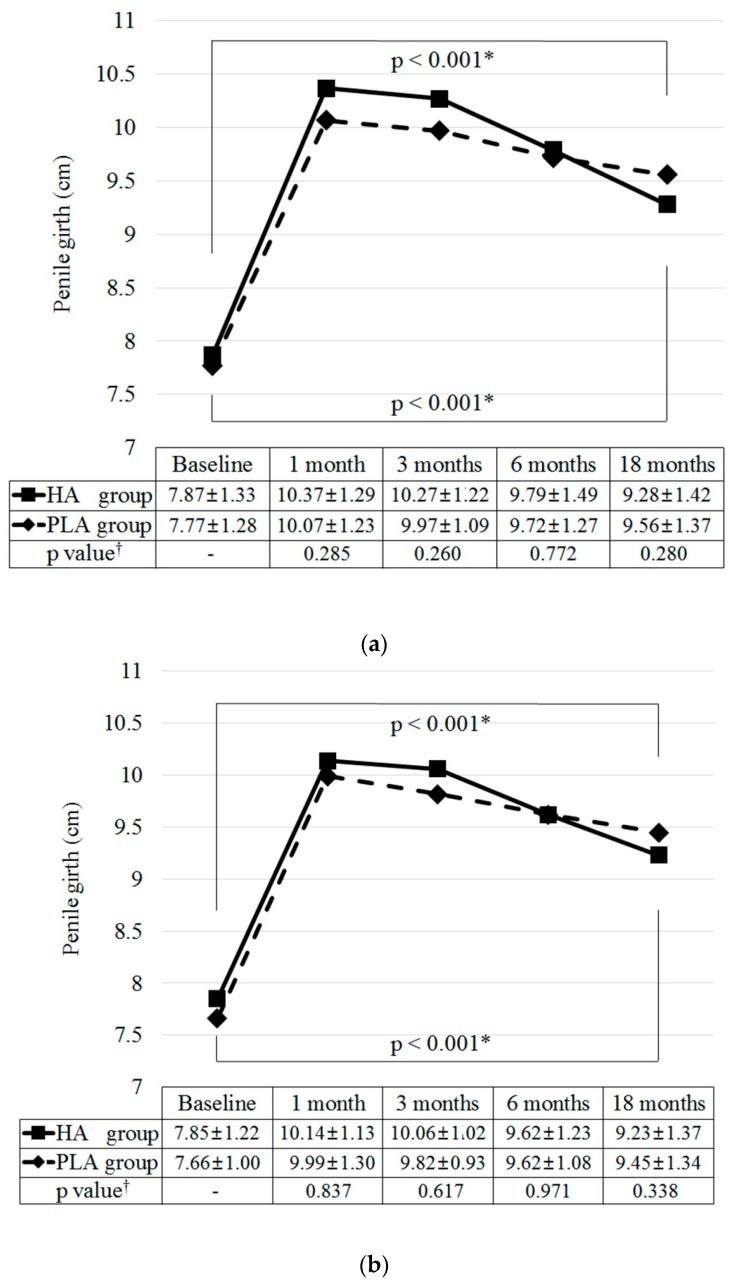

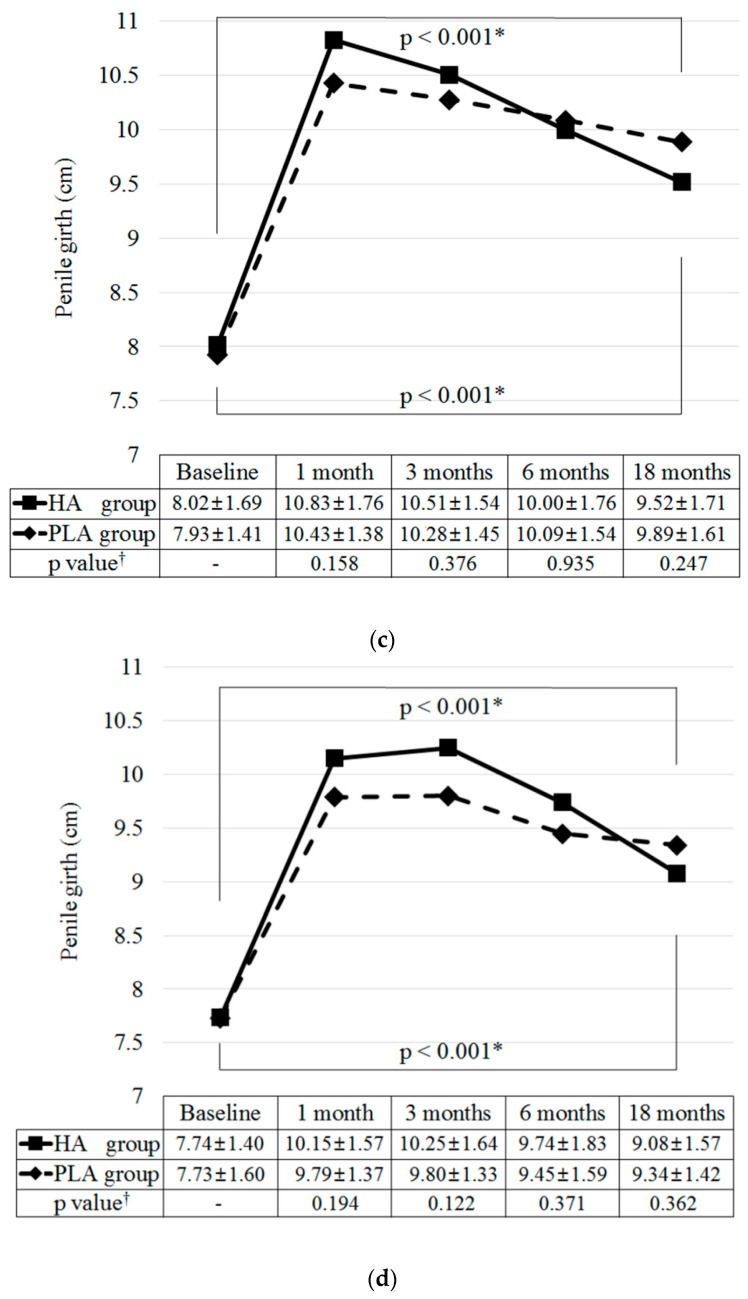
Changes of penile girth before and after hyaluronic acid and polylactic acid filler injections: (**a**) mean penile girth, (**b**) distal shaft, (**c**) mid-shaft, (**d**) proximal shaft. The mean penile girth was defined as the mean of the circumference measures on distal, mid-, and proximal shafts. * Paired t-test. † Injected filler volume and baseline penile girth-adjusted analysis of covariance test from baseline. HA, hyaluronic acid; PLA, polylactic acid.

Figure 3 shows the changes in penile girth before and after filler injections. In both groups, the mean penile girths increased to a maximum at 1 month (mean increase of 2.50 ± 0.88 cm in the HA group, *p* < 0.001; mean increase in the 2.30 ± 0.98 cm in the PLA group, *p* < 0.001) (Figure 3a). Then, the mean girths in the HA group gradually decreased for up to 18 months (−1.09 ± 1.51 cm from 1 month to 18 months; *p* < 0.001), whereas those in the PLA group lasted up to 18 months (−0.51 ± 1.66 cm from 1 month to 18 months, *p* = 0.123). However, the mean penile girths were significantly increased at 18 months compared to the baseline in both groups (mean increase of 1.41 ± 1.48 cm in the HA group, *p* < 0.001; mean increase of 1.79 ± 1.41 cm in the PLA group, *p* < 0.001). During the study period, the changes in the mean penile girth in the HA group were not statistically different from those in the PLA group (*p* = 0.285, *p* = 0.260, *p* = 0.772, and *p* = 0.280 at 1, 3, 6, and 18 months after injection). However, from 1 month to 18 months, the reduction in the augmentative effect in the HA group was greater than that in the PLA group (43.6% vs. 22.1%; *p* = 0.015). The trends in results measured at the distal, mid-shaft, and proximal shaft were similar to those of the mean penile girth (Figure 3b–d).

### 3.3. Satisfaction Level

**Figure 4:**
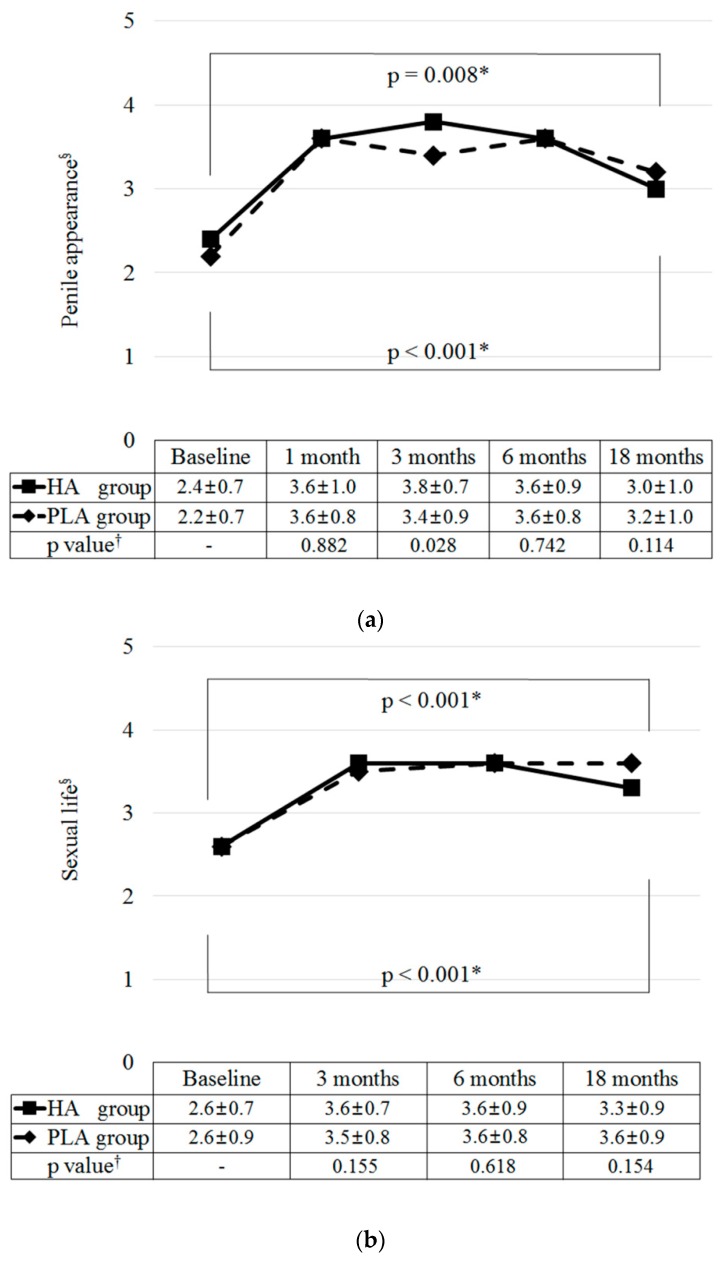
Changes of satisfaction associated with hyaluronic acid and polylactic acid filler injections: (**a**) penile appearance, (**b**) sexual performance. * Paired t-test. ^†^ Injected filler volume and baseline penile girth-adjusted analysis of covariance test from baseline. ^§^ Satisfactions were assessed with a 5-point visual analogue scale, with 1 indicating very dissatisfied and 5 indicating very satisfied. HA, hyaluronic acid; PLA, polylactic acid.

At one month, the VAS scores for penile appearance significantly increased in both groups (mean increase of 1.2 ± 1.1 in the HA group, *p* < 0.001; mean increase of 1.4 ± 1.0 in the PLA group, *p* < 0.001) (Figure 4a). These increases lasted until six months (*p* = 0.677 from one month to six months in the HA group; *p* = 0.572 in the PLA group). After that time, they gradually decreased until 18 months (*p* = 0.007 from 6 to 18 months in the HA group; *p* = 0.039 in the PLA group). However, the VAS scores at 18 months were significantly higher than those at baseline in both groups (*p* = 0.008 and *p* < 0.001 in the HA and PLA groups, respectively). Changes in the VAS scores for penile appearance did not differ significantly between groups during the study period (*p* = 0.273, 0.882, 0.028, 0.742, and 0.114 at baseline, 1, 3, 6, and 18 months, respectively).

At three months, the VAS scores for sexual performance significantly increased in both groups (mean increase of 1.1 ± 0.9 in the HA group, *p* < 0.001; mean increase of 0.9 ± 1.2 in the PLA group, *p* < 0.001) (Figure 4b). These increases lasted up to 18 months (*p* = 0.065 from 3 to 18 months in the HA group; *p* = 0.802 in the PLA group). Changes in the VAS scores for sexual performance did not differ significantly between groups during the study period (*p* = 0.930, 0.155, 0.618, and 0.154 at baseline, 3, 6, and 18 months, respectively).

### 3.4. Safety

Immediately after injections, penile swelling or bruising was observed, but disappeared within a week. The AEs associated with injections were reported for three (9.1%) patients in the HA group and two (5.9%) patients in the PLA group; there was no significant difference between groups (*p* = 0.972) (Table 2). All AEs were mild and cured with NSAIDs during the study period. No serious AEs were reported during the study period. Additionally, significant changes were not observed in the physical examination results, vital signs, and laboratory examination results.

## 4. Discussion

Our study showed that HA and PLA fillers injected in the penis led to significant augmentation of the penile girth and to increased injection-associated satisfaction. The injections were tolerable, and no serious AEs occurred for up to 18 months. Clinical efficacy and safety were comparable between groups. To our knowledge, this is the first randomized comparative trial to use 18 months of follow-up to compare the clinical courses of HA and PLA fillers for PA.

Many men are dissatisfied with their penile size and desire a larger penis [17]. In a modern study of 25,594 men, 45% indicated that they desired a larger penis [5]. Therefore, various types of surgical and non-surgical PA methods have been introduced [6]. However, these PA methods do not have clearly proven efficacy and safety, and they have not been globally approved [18].

Soft tissue filler injections have become the main treatment for soft tissue augmentation [2]. In the field of PA, several types of fillers have been introduced, and a few are available on the market [8,14]. As different types of fillers have varying biochemical and clinical characteristics, it is critical for physicians to understand the clinical course after injections of different fillers [8,15]. Both HA and PLA fillers, which were used in our study, are regarded as temporary, and they are approved for PA in many countries [15]. However, these two fillers have different biochemical characteristics. HA comprises natural ingredients and has direct and passive effects [8]. After injection of HA filler, it is gradually absorbed by the surrounding tissue. During degradation, as molecules bind more water, the same volume can be maintained with less HA for a few months. Then, volume decreases steadily. PLA is a synthetic polymer that has delayed and bio-stimulatory effects [8]. After injection, liquid in PLA filler becomes absorbed over a few days. For a few months, microparticles of PLA filler become surrounded in a connective tissue capsule and are gradually degraded by hydrolysis, while the injected site undergoes subtle volume expansion by a fibrous tissue response with collagen deposition. Then, volume decreases steadily. The augmentative effect of HA filler lasts up to 18 months; however, that of PLA filler lasts for up to three years [8,10,11,19].

To our knowledge, there are only two randomized trials about the comparison of clinical utility between the fillers for PA [7,15]. One assessed 36 men receiving HA filler and 36 receiving PLA filler during a 48-week period after injection [7]. At 48 weeks after injection, the penile girth increases were approximately 1.7 cm and 1.3 cm in the HA and PLA groups, respectively. There were no significant differences in the changes in penile girth and satisfaction associated with them. The other study assessed 39 men receiving HA filler and 35 receiving PLA filler during a 24-week period after injection [15]. At 24 weeks after injection, the penile girth increases were approximately 2.1 cm and 1.6 cm in the HA and PLA groups, respectively. There were no significant differences in the changes in penile girth and satisfaction associated with them. The results of these two previous trials are similar to the present study proving the clinical utility of HA and PLA fillers for PA, and they also showed comparable efficacy and safety between the two fillers. However, our randomized comparative trial involved a longer follow-up period (18 months) to assess PA. As the HA and PLA fillers are temporary, long-term follow-up after injection is of paramount importance.

Our study and the previous two studies have also shown similar clinical courses after the HA and PLA filler injections [7,15]. In terms of augmentative effects, the penile girth after HA filler injection increased to a maximum within approximately one month. On the other hand, the girth after PLA filler injection increased to a maximum at approximately one to three months. However, these girth increases lasted up to 18 months after injections of both HA and PLA fillers. A comparison of the two fillers indicated that the decreasing slope of increased girth tended to be greater for HA filler than for PLA filler. These clinical patterns could be explained by the different biochemical characteristics of the two fillers. Satisfaction with penile appearance significantly increased within approximately one month after both filler injections. These increases lasted approximately 6 to 12 months. Then, they gradually decreased until 18 months; however, the satisfaction levels were still higher than those at baseline. Changes in satisfaction with penile appearance did not seem to differ significantly between groups during the 18 months. As almost all filler injections for PA are performed due to the desires of men reporting a small penis, it is very important to understand the different clinical courses of various fillers after injection. Therefore, our study might help physicians choose the most appropriate type of filler.

Interestingly, our study and the previous two studies have demonstrated that increased satisfaction with sexual performance after both filler injections lasted 18 months, even as the increased penile girth gradually decreased with time [7,15]. This finding suggests that the filler injection for PA might alleviate the psychological distress associated with sexual performance [15]. It also supports the idea that men who want to enlarge their penis have psychological distress that could require counseling or psychotherapy [18].

Notably, the injection of filler should be performed with caution. After injection, the penis cannot be completely restored to its pre-injection state. Furthermore, there are possible side effects and complications associated with filler injections. Therefore, it is critical to counsel men seeking PA to provide them with details about the realistic effects and possible side effects. An interesting finding of one study of men reporting a small penis was that only 3.6% chose to seek PA after undergoing structured management and counseling [20].

Two cases of injection site inflammation and three cases of injection site pain were reported in our study. All cases were mild and treated with NSAIDs. These results were comparable with those of previous studies [7,10,11,15], and both fillers seemed safer than surgery [14,21,22].

Our study has a few limitations. First, an operator-blinded trial was impossible because of the unique characteristics of each filler material. Instead, all assessments after injections were performed by trained independent physicians who were not involved in the screening or filler injection. Second, the VAS was used to assess the satisfaction levels. However, additional tools for assessing sexual and psychological distress might provide more information associated with the choice to undergo filler injections for PA. Finally, the erect penile girth was not measured because of the technical difficulty. However, the size of the flaccid girth (compared to that of the erect girth) was likely the cause of distress for men reporting a small penis [6,20]. In addition, no erection-associated problems were reported.

## 5. Conclusions

For men reporting a small penis, HA and PLA filler injections for PA led to significant augmentation of the penile girth and increased satisfaction for up to 18 months. Clinical efficacy and safety were comparable for both fillers. However, there were some differences in the clinical courses of HA and PLA fillers. Our study suggests that filler injections can be cautiously used to treat men who want a larger penis. It also might help physicians choose the most appropriate type of filler for each patient.

## Figures and Tables

**Table 1:** Baseline demographic and clinical characteristics.

Variables	All (N = 67)	HA Group (N = 33)	PLA Group (N = 34)	*P* Value
Age, years, mean (SD)	42.9 (7.9)	41.9 (6.3)	43.8 (9.1)	0.353 *
Weight, kg, mean (SD)	75.1 (8.8)	75.6 (9.5)	74.6 (8.2)	0.636 *
Height, cm, mean (SD)	172.3 (5.1)	172.6 (4.7)	171.9 (5.6)	0.597 *
Diabetes mellitus, n (%)	4 (6.0)	2 (6.1)	2 (5.9)	1.000 **
Hypertension, n (%)	4 (6.0)	2 (6.1)	2 (5.9)	1.000 **
Dyslipidemia, n (%)	4 (6.0)	0 (0.0)	4 (11.8)	0.114 **
Benign prostatic hyperplasia, n (%)	7 (10.4)	1 (3.0)	6 (17.6)	0.105 **
Erectile dysfunction, n (%)	5 (7.5)	1 (3.0)	4 (11.8)	0.356 **
Premature ejaculation, n (%)	0 (0.0)	0 (0.0)	0 (0.0)	-
Circumcision, n (%)	1 (1.5)	0 (0.0)	1 (2.9)	1.000 **
Penile girth, cm, mean (SD)				
mean ^†^	7.82 (1.30)	7.87 (1.33)	7.77 (1.28)	0.763 *
distal shaft	7.75 (1.11)	7.85 (1.22)	7.66 (1.00)	0.480 *
mid-shaft	7.97 (1.54)	8.02 (1.69)	7.93 (1.41)	0.822 *
proximal shaft	7.74 (1.50)	7.74 (1.40)	7.73 (1.60)	0.975 *
Satisfaction level, mean (SD) ^††^				
penile appearance	2.3 (0.7)	2.4 (0.7)	2.2 (0.7)	0.273 *
sexual performance	2.6 (0.8)	2.6 (0.7)	2.6 (0.9)	0.930 *

HA, hyaluronic acid; PLA, polylactic acid; n, number of patients; SD, standard deviation. * Student’s *t* test. ** Fisher’s exact test. ^†^ The mean penile girth was defined as the mean of the circumference measures on distal, mid-, and proximal shafts. ^††^ Satisfactions were assessed with a 5-point visual analogue scale, with 1 indicating very dissatisfied and 5 indicating very satisfied.

**Table 2:** Filler injection-related adverse events.

Adverse Events	HA Group (N = 33)			PLA Group (N = 34)		
	Cases	Degree	Recovery	Cases	Degree	Recovery
Injection site inflammation	1 (3.0%)	Mild	Yes	1 (2.9%)	Mild	Yes
Injection site pain	2 (6.1%)	Mild	Yes	1 (2.9%)	Mild	Yes

HA, hyaluronic acid; PLA, polylactic acid.
